# Stem cell-derived extracellular vesicles enriched with miR-22-3p for cataract treatment—Molecular mechanisms and therapeutic potential: A review

**DOI:** 10.17305/bb.2026.13941

**Published:** 2026-04-17

**Authors:** Xingcheng Li, Lili Zhang

**Affiliations:** 1 Department of Fundus Disease Specialty, Lanzhou Aier Eye Hospital, Lanzhou, China; 2 Department of Glaucoma, Lanzhou Aier Eye Hospital, Lanzhou, China

**Keywords:** Cataracts, extracellular vesicle, *miR-22-3p*, neuroprotective mechanisms, stem-cell therapy

## Abstract

Cataracts, characterized by progressive lens opacification, affect a significant number of individuals globally, particularly the elderly and those with systemic diseases. Research on the pathogenesis of visual impairments has identified alterations in crystalline proteins, primarily driven by inflammation, apoptosis, oxidative stress, and fibrosis in lens epithelial cells, as the primary mechanisms contributing to cataract development. Current treatment options, including surgical lens implantation and stem cell therapy, have notable limitations, highlighting the need for alternative non-invasive therapies. Stem cell-derived extracellular vesicles (SC-EVs) have emerged as a novel therapeutic approach for various diseases. This review aims to comprehensively analyze the literature regarding the therapeutic potential of SC-EVs in cataract treatment, particularly when enriched with *miR-22-3p*. A literature search was conducted focusing on the molecular mechanisms of *miR-22-3p* in lens epithelial cells and cataract models, discussing its regulatory pathways in retinal and optic nerve injuries, as well as the advantages of utilizing SC-EVs as nanocarriers for targeted therapy. Studies indicate that *miR-22-3p* can inhibit crystalline remodeling by modulating gene expression and regulating apoptosis, fibrosis, oxidative stress, and inflammation. Consequently, it plays a role in the signaling pathways associated with cataracts and may also exhibit neuroprotective effects in neurons and retinal ganglion cells. By integrating current insights from research on cell-free EV therapies and the molecular mechanisms of *miR-22-3p* in cataract pathology, this review underscores the potential of *miR-22-3p*-enriched SC-EVs as a promising therapeutic option for cataracts. However, this hypothesis necessitates rigorous preclinical and clinical validation.

## Introduction

Cataracts are a leading cause of visual impairment and blindness worldwide, primarily resulting from age-related lens opacification due to the accumulation and modification of proteins in the crystalline lens, oxidative stress imbalance, and damage to ocular cells [1]. Different types of cataracts include fibrotic cataracts, which are secondary to metabolic disorders such as diabetes, trauma, and prolonged exposure to ultraviolet irradiation [1]. While surgical replacement with an artificial intraocular lens is the current standard treatment, it is not universally applicable. Moreover, the risk of postoperative complications—including posterior capsule opacification, infection, inflammation, and fibrosis—underscores the urgent need for alternative, ideally non-invasive treatment strategies that can prevent cataract-related complications [2]. Various pharmaceutical approaches, such as antioxidants, photodynamic therapy, and other minimally invasive interventions, have been previously investigated [3, 4].

Stem cell therapy for cataracts is an innovative therapeutic option that offers advantages such as the potential for natural lens regeneration, improved vision, and fewer complications compared to conventional surgery, along with immunomodulation in tissue repair. However, challenges persist, including technical complexity, the risk of immune rejection, tumorigenesis, and variable effectiveness depending on disease severity [5].

Extracellular vesicles (EVs) have emerged as significant therapeutic options for a range of eye diseases, including corneal injuries, glaucoma, and retinal degeneration [6, 7]. EVs serve as nanocarriers for diverse cargo, including bioactive compounds such as lipids, proteins, and nucleic acids. Stem cell-derived extracellular vesicles (SC-EVs) are particularly valuable due to their multipotent nature, which enhances the functionality of ocular cells and facilitates targeted drug delivery [6].

Nucleic acids, especially non-coding RNAs, are key functional bioactive molecules present in EVs [8]. Micro RNAs (miRNAs) are small non-coding RNAs that regulate post-transcriptional gene expression. Recent studies comparing miRNA expression profiles in healthy individuals to those with various physiological and pathological conditions have underscored their significance as functional biomolecules in the diagnosis and monitoring of targeted therapeutic strategies. Notably, *miR-22-3p* has emerged as a crucial regulator in the pathogenesis of cataracts, playing multifaceted roles in modulating inflammation, apoptosis, oxidative stress, and cellular anti-fibrotic and neuroprotective processes (Figure 1) [9, 10]. Previous studies have reported downregulation of *miR-22-3p* in senile and diabetic cataracts, where it targets Kruppel-like factor (KLF6) to prevent hyperglycemia-induced apoptosis in lens epithelial cells (LEC) [10]. Furthermore, *miR-22-3p* exhibits neuroprotective effects, inhibits inflammation and oxidative damage to LEC, and prevents fibrosis by suppressing epithelial-mesenchymal transition.

**Figure 1.**
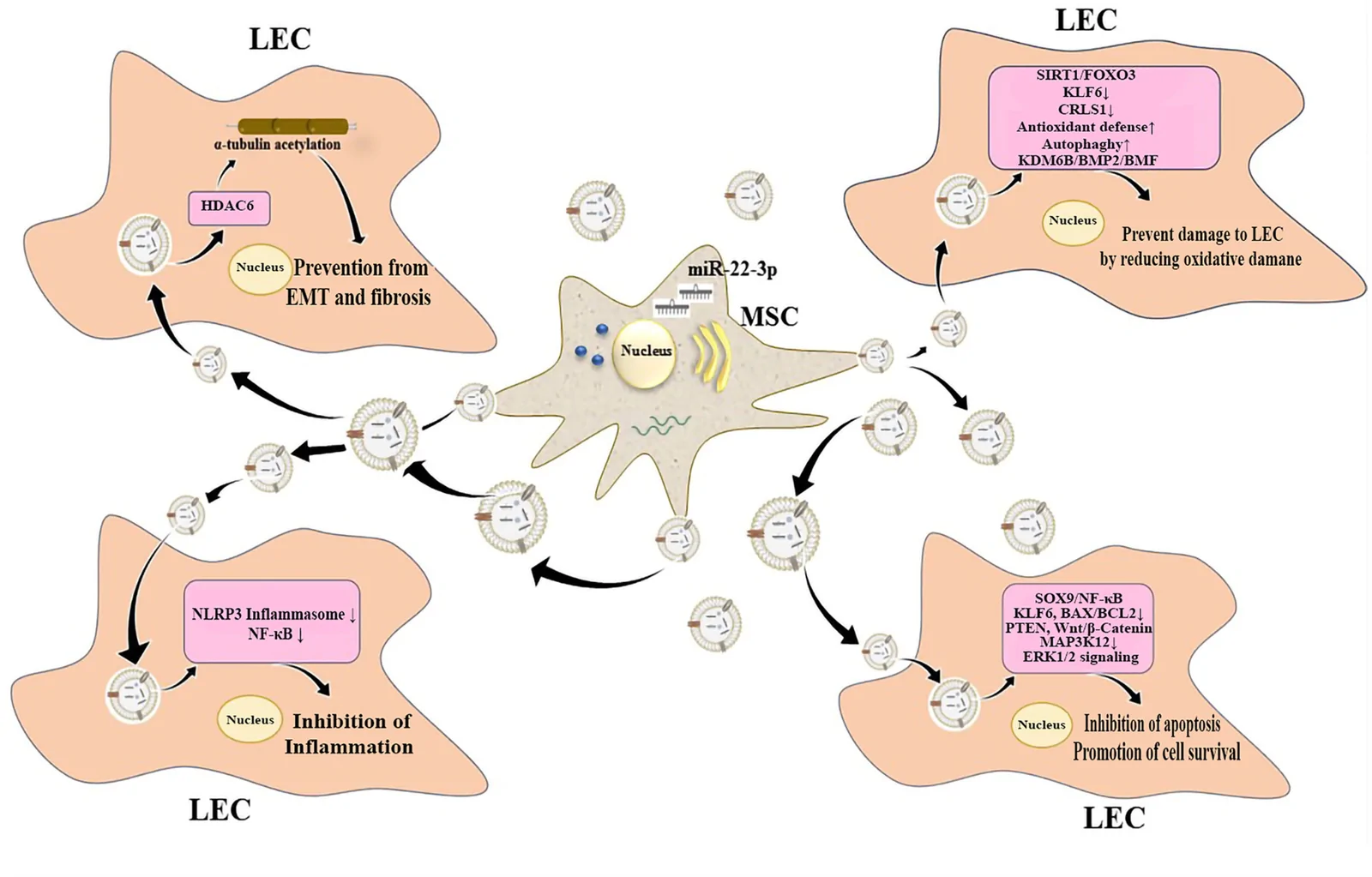
**Neuroprotective effects of *miR-22-3p*-enriched MSC-EVs on lens epithelial cells.** This figure illustrates the potential therapeutic applications of *miR-22-3p*-enriched MSC-EVs in the treatment of cataracts. The functions attributed to *miR-22-3p* in LECs include the inhibition of epithelial-mesenchymal transition, anti-fibrotic effects, enhancement of antioxidant defenses and autophagy, modulation of cell survival, and anti-apoptotic activities. These effects can be significantly enhanced through encapsulation within MSC-EVs. **Abbreviations**: BAX, BCL-2-associated X protein; BCL-2, B-cell lymphoma 2; CRLS1, cardiolipin synthase gene 1; ERK1/2, extracellular signal-regulated kinases 1/2; HDAC6, histone deacetylase 6; KDM6B/BMP2/BMF, histone lysine demethylase 6B/bone morphogenetic protein 2/bone marrow failure; KLF6, Kruppel-like factor 6; LECs, lens epithelial cells; MAPK12, mitogen-activated protein kinase 12; miR-22-3p, microRNA-22-3p; MSC-EVs, mesenchymal stem cell-derived extracellular vesicles; NF-κB, nuclear factor-kappa B; NLRP3, NOD-like receptor family pyrin domain containing 3; PTEN, phosphatase and tensin homolog; SIRT1/FOXO3, sirtuin 1/Forkhead box O3a; SOX9, SRY-related high-mobility group box 9; Wnt/β-catenin, wingless-related integration site/β-catenin.

One concept posits that *miR-22-3p* can be enriched within EVs and exert enhanced effects when directly transferred to target cells. Therefore, this study comprehensively reviews published research on the role of *miR-22-3p* in lens biology and cataract pathogenesis, as well as the therapeutic potential of *miR-22-3p*-enriched SC-EVs. It emphasizes the advantages of employing SC-EVs for drug delivery and elucidates the functional mechanisms of *miR-22-3p* in cellular processes under both physiological and pathological conditions. Although the primary focus is on cataracts, this review also discusses the broader neuroprotective profile of *miR-22-3p* in ocular and central nervous system models, suggesting innovative combination strategies for enhancing lens transparency and protecting visual pathways in complex ocular diseases. By integrating current knowledge from molecular biology, stem cell research, and therapeutic strategies, this review provides foundational insights for future translational research and potential clinical applications aimed at reducing the societal burden of cataracts through miRNA-based SC-EV therapies.

## Methodology and literature selection

A literature search was conducted to identify publications from 2008 to 2025 regarding the role of *miR-22-3p* in lens biology and cataract pathogenesis, as well as the therapeutic potential of SC-EVs in ocular diseases. This search utilized online databases, including PubMed, Web of Science, Scopus, and Embase, employing specified search keywords and Boolean operators. The keywords included: for cataracts and lens epithelial cells (“cataract,” “lens epithelial cell,” “lens opacification”); for *miR-22-3p* (“*miR-22-3p*,” “*miR-22*,” “miRNA,” “microRNA,” and “non-coding RNA”); and for EVs and stem cell components (“extracellular vesicle,” “exosome,” “stem cell,” “mesenchymal stem cell”), combined with Boolean operators (AND, OR, NOT).

Studies were deemed eligible if they were written in English as experimental or clinical original articles or relevant review articles that aligned mechanically with the aims and scope of our study. Editorials, commentaries, irrelevant papers, and non-English conference abstracts without full texts were excluded.

## SC-EVs

### EVs as key nanocarriers in cellular communication

EVs are heterogeneous bilayer-membrane-coated vesicles naturally produced by various cell types, present in the extracellular matrix, and essential for mediating intracellular communication. Their most significant feature is cell-to-cell communication, which facilitates the transmission of important cargo, such as lipids, proteins, messenger RNAs, and non-coding RNAs, originating primarily from their parent cells. EVs can also transport cytokines, chemokines, cell-specific antigens, cytoplasmic enzymes, heat shock proteins, and molecules associated with cell adhesion. They vary in size, cargo type, and biogenesis, classified into three main categories: exosomes, microvesicles, and apoptotic bodies. Exosomes, typically the smallest EVs (30–150 nm in diameter), originate from late endosomes and facilitate cargo transfer by fusing with target cell membranes. Microvesicles and apoptotic bodies are larger (100–1000 and >1000 nm, respectively) and originate from exocytosis or dying cells. Among these, small EVs, including exosomes and microvesicles, have garnered significant interest in stem cell therapy due to their potential as therapeutic agents [11].

### Unique features of stem-cell-derived EVs in ocular therapy

In recent years, the use of EVs has emerged as a promising cell-based approach in preclinical ocular models, with several studies reporting favorable treatment outcomes. Owing to their small size, EVs are bioavailable nanocarriers that can easily traverse natural barriers in the body, enabling effective treatment at lower doses—an essential aspect of precision medicine [11]. SC-EVs have attracted considerable interest among researchers as promising therapeutic mediators. These nanovesicles are typically secreted by various stem cells, such as mesenchymal stem cells (MSCs). MSCs, non-hematopoietic stem cells of mesodermal origin, exhibit a fibroblast-like appearance and grow clonally. They have been discovered in tissues such as subcutaneous fat, muscle, lungs, liver, pancreas, umbilical cord blood, and breast milk. MSCs are extensively studied due to their anti-inflammatory, regenerative, and immunoregulatory properties. Despite the limitations of stem cell transplantation—such as the risk of immune rejection, tumorigenesis, and poor survival of transplanted cells—research on SC-EVs has demonstrated advantages over direct cell transplantation, primarily by avoiding immune responses and the potential transfer of mutated or cancerous cells [12].

In ocular applications, the non-invasive administration of SC-EVs topically or through minimally invasive methods such as intravitreal or periocular injections has proven effective in enhancing the bioavailability of their components in target tissues. MSC-derived EVs (MSC-EVs) are particularly promising in clinical studies, as they originate from parental MSCs known for their high proliferative capacity and low immunogenicity. MSC-EVs are natural, cell-free structures that can be widely utilized without the limitations associated with MSCs, serving as effective carriers for proteins, lipids, nucleic acids, and medications, thereby facilitating targeted delivery and immunological escape in therapeutic approaches.

### SC-EVs as promising therapeutic agents in ocular diseases

In recent years, EVs have emerged as transformative agents in the research and treatment of ocular diseases. The therapeutic effects of EVs have been studied in conditions such as corneal injury, glaucoma, optic nerve atrophy, diabetic retinopathy, and lens epithelial cataracts [13]. MSC-EVs in a retinal injury mouse model improved neuronal survival, stimulated angiogenesis, and reduced inflammation [14]. In a study involving human corneal endothelial cells, Yu et al. demonstrated the wound-healing ability of MSC-EVs and their role in enhancing the regeneration of corneal endothelial cells [15].

Li et al. investigated the neuroprotective effects of EVs derived from umbilical cord MSCs in a rat model of optic nerve injury [16]. They initially established glaucoma-like optic nerve damage in the model and then intravitreally injected umbilical cord MSC-EVs. Following an eight-week period, they evaluated treatment efficacy on retinal damage, survival of retinal ganglion cells, and expression levels of caspase-3 as an apoptotic biomarker. Their observations of reduced cell apoptosis and mitigation of optic nerve damage validated the protective effects of umbilical cord MSC-EVs as cell-free therapeutic agents in retinal ganglion cell damage [17].

Kim et al. explored the therapeutic effects of SC-EVs on retinal ganglion degeneration in an animal mouse model. They emphasized the disadvantages and limitations of direct stem cell transplantation, introducing SC-EVs as having neuroprotective effects superior to other treatments due to their miRNA cargoes with neuroprotective and anti-inflammatory effects [18].

### EVs as natural carriers of miRNAs to their target cells

SC-EVs play a pivotal role in mediating essential effects across a range of ocular diseases, including cataracts. They facilitate the delivery of regulatory molecules, such as effector proteins and miRNAs, which contribute to anti-inflammatory, anti-apoptotic, and protective responses under oxidative stress conditions. Additionally, SC-EVs are characterized by their ability to activate microglial cells, inhibit inflammasome pathways, and promote tissue regeneration. Most SC-EVs exert therapeutic effects through paracrine signaling mechanisms by encapsulating signaling molecules, particularly miRNAs, which are critical for various cellular processes including apoptosis, inflammation, proliferation, and tissue regeneration (Table 1).

**Table 1:** Therapeutic and functional effects of various mirnas in cataract research

**Study**	**Study model**	**Cataract subtype**	**miRNA**	**Directionally**	**Target/pathway**	**Key functional conclusion**	**Reference**
			*miR-124*, *miR-125a*	Upregulated			
Zhang et al., 2025	Meta analysis and bioinformatics	Age-related cataracts	*miR-23b*, *miR-15a*, *miR-221*, *miR-222*, *miR-378a*, *miR-34a*	Downregulated	Not specified	Biomarker identification	[40]
Sun et al., 2023	*in vivo* and *in vitro*	Age-related cataracts	*miR-29b*	Downregulated	Smac/DIABLO (via increasing TUG1)	Modulation of apoptosis through TUG1/miR-29b/Smac axis	[41]
Li et al., 2019	LEC B3 cell line	Age-related cataracts	*miR-15a*	Not specified	BCL-2 and E2F3	modulating proliferation and apoptosis in LEC	[22]
Zhou et al., 2019	LEC SRA01/04 cell line (oxidative stress model)	Age-related cataracts	*miR-23b-3p*	Not specified	SIRT1	targeting regulates apoptosis and autophagy in LEC	[19]
Bao et al., 2024	Bioinformatic; LEC SRA01/04 cell line	Age-related cataracts	*miR-492*	Not specified	nucleoplasmin-1 (NPM1) (TGF-β2/*miR-492*/ NPM1axis)	Regulates apoptosis, proliferation, EMT, and autophagy	[21]
Ma et al., 2025	*In vivo* and *in vitro*	Fibrotic cataracts	*miR-148a-3p (exosomal)*	Upregulated	Not specified	inhibits EMT	[42]
Wang et al., 2024	mouse model (*in vivo*)	Anterior subcapsular (fibrotic) cataracts	*miR-1225-5p*	Not specified	*miR-1225-5p*/keap1/Nrf2 signaling axis	Induces the lens fibrosis.	[43]
Guo et al., 2023	*In vitro* (oxidative stress)	Oxidative stress-induced age-related cataracts	*miR-124-3p*	Not specified	DAPK1 (*NEAT1*/*miR-124-3p*/DAPK1 signaling)	LEC apoptosis and cataracts formation	[44]
Fang et al., 2023	Rat cataract model; (Transcriptome sequencing *In vivo* and *in vitro*)	Cataracts (subtype not specifies)	*miR-6848-5p*	Not specified	ALOX15 *(Circ 06209* sponge *miR-6848-5p* /ALOX15 pathway)	prevents cataract (necroptosis-related)	[45]
Yao et al., 2021	*In vitro* LEC	Not specified	*miR-23a-3p*	Not specified	BCL-2	Regulate cell proliferation and apoptosis	[46]

**Abbreviations**: ALOX15, arachidonate 15-lipoxygenase 15; BCL-2, B-cell lymphoma 2; DAPK1, death-associated protein kinase 1; E2F3, E2F transcription factor 3; EMT, epithelial–mesenchymal transition; Keap1, Kelch-like ECH-associated protein 1; LEC, lens epithelial cells; miRNA, microRNA; NEAT1, nuclear paraspeckle assembly transcript 1; NPM1, nucleophosmin 1; Nrf2, nuclear factor erythroid 2-related factor 2; SIRT1, sirtuin 1; Smac/DIABLO, second mitochondria-derived activator of caspases/direct IAP-binding protein with low pI; TGF-β2, transforming growth factor beta 2; TUG1, taurine upregulated gene 1.

miRNAs represent a major category of cargo within EVs, capable of transferring to recipient cells and exerting regulatory effects. The functional role of exosomal miRNAs in ocular diseases has been extensively studied. For instance, Zhou et al. investigated age-related cataracts under oxidative stress and identified that *miR-23b-3p* directly targets sirtuin 1 (SIRT1) in lens epithelial cells, thereby regulating apoptosis and autophagy [19]. Xia et al. explored the functional role of exosomal *miR-29b*, which is involved in the calcium signaling pathway in diabetic cataract patients. They proposed exosomal *miR-29b* or its targeted messenger RNAs as potential therapeutic targets for treating diabetic cataracts [20]. Moreover, *miR-492* targets nucleoplasmin-1 (NPM1) and modulates the transforming growth factor beta 2 (TGF-β2)/*miR-492*/NPM1 axis, influencing apoptosis, proliferation, epithelial-mesenchymal transition, and autophagy in lens epithelial cells affected by age-related cataracts [21]. Li et al. demonstrated that *miR-15a* directly targets B-cell lymphoma 2 (BCL-2) and E2F transcription factor 3 (E2F3), thereby modulating proliferation and apoptosis in lens epithelial cells associated with age-related cataracts [22]. In a comparative study on lens capsule epithelium, Kim et al. analyzed miRNA expression patterns across various cataract subtypes and healthy volunteers. Their findings revealed downregulation of *let-7a-5p*, *miR-22-3p*, *let-7d-5p*, and *miR-16-5p*, alongside upregulation of *miR-23a-3p*, *miR-23b-3p*, *miR-125a-5p*, and *let-7g-5p* in cataract patients. These results underscore the significance of miRNA expression profiles in identifying non-invasive biomarkers relevant to pathogenesis, diagnosis, and targeted therapy [23].

## Molecular and neuroprotective function of miR-22-3p

*miR-22-3p* is a tissue-specific small non-coding RNA that functions as a post-transcriptional regulator by binding to the 3′ untranslated region of target messenger RNAs. It is transcribed from the 13th locus on the short arm of chromosome 17 (17p13.3), with documented expression in the brain, heart, glia, neurons, and lens epithelial cells. The functional significance of *miR-22-3p* is underscored by its role in regulating gene expression and modulating fundamental cellular processes, including hypoxia, apoptosis, inflammation, and oxidative stress responses.

### Functional role of miR-22-3p in lens epithelial cells and cataract models (direct evidence)

This section summarizes findings from studies that have directly investigated lens epithelial cells and cataract models (both *in vitro* and *in vivo*), distinguishing them from retinal, optic nerve, and other non-lens neuronal models. These studies provide direct mechanistic insights into the role of *miR-22-3p* in lens epithelial cells and cataract models.

#### *miR-22-3p* and apoptosis

*miR-22-3p* plays a role in anti-apoptotic mechanisms by regulating the expression of pro-apoptotic proteins, such as phosphatase and tensin homolog (PTEN), B-cell lymphoma (BCL), BCL-2-associated X (BAX), B-cell lymphoma 2 interacting mediator (BIM), and p53. Consequently, it influences signaling pathways associated with cellular apoptosis. Yin et al. reported that downregulation of *miR-22-3p* under hyperglycemic conditions correlates with increased expression of KLF6, a transcription factor that induces apoptosis in lens epithelial cells, thereby contributing to cataract formation [10]. Xu et al. proposed the anti-apoptotic role of *miR-22-3p* in a study on retinal ischemia, indicating that intervention to restore *miR-22-3p* levels can rebalance the expression of anti- and pro-apoptotic proteins such as BCL-2 and BAX [23]. Collectively, these studies suggest that modulation of *miR-22-3p* may influence apoptosis in lens epithelial cell and cataract models.

#### *miR-22-3p* and oxidative stress

Oxidative stress is a significant risk factor for cataract formation, involving mechanisms such as oxidative damage to lens epithelial cells, reduced cellular antioxidant capacity, and alterations in lens crystallin proteins. The accumulation of reactive oxygen species (ROS)—from external sources (e.g., smoking, air pollution, sunlight) or generated endogenously due to aging or pathological conditions (e.g., hypertension, diabetes)—can diminish the total antioxidant capacity of lens epithelial cells. Key mechanisms include oxidative damage to mitochondrial and endoplasmic reticulum functions, activation of Sirtuin 1/Forkhead box O3a (SIRT1/FOXO3) and Nuclear factor erythroid 2-related factor 2/antioxidant response element (Nrf2/ARE) pathways, induction of cell death, and impairment of gap junctions. *miR-22-3p* acts as a potent antioxidant modulator by targeting cardiolipin synthase gene-1 (*CRLS1*), a key regulator of antioxidant activity, thereby influencing mitochondrial function [24]. Hu et al. demonstrated that overexpression of *miR-22-3p* correlates with reduced oxidative damage in ocular epithelial cells, decreased ROS levels, and lowered secretion of inflammatory cytokines [25]. Furthermore, *miR-22-3p* downregulates KLF6 expression, which is critical for promoting cell death under hyperglycemic-induced oxidative stress conditions [10]. Given that these mechanisms have been demonstrated in lens epithelial cells, they provide direct evidence supporting the potential anti-inflammatory and antioxidant properties of *miR-22-3p* in cataract pathology.

#### *miR-22-3p* and inflammation

Downregulation of *miR-22-3p* is associated with the activation of inflammatory pathways, cytokine production, and subsequent fibrosis and cell death. Kim et al. reported decreased levels of *miR-22-3p* in patients with senile cataracts, significantly correlating with increased oxidative stress and impaired autophagy in ocular cells [18]. The anti-inflammatory mechanisms of *miR-22-3p* may involve targeting NOD-like receptor family pyrin domain-containing 3 (*NLRP3*) messenger RNA, a key mediator of inflammation in retinal pigment epithelial cells, which is activated under high ROS levels and leads to overproduction of inflammatory cytokines such as caspase-1, IL-1β, and IL-18 [26, 27].

#### *miR-22-3p* as a protective factor under hypoxia

The hypoxia-inducible factor-1α (HIF-1α) signaling pathway contributes to cataract pathogenesis by promoting epithelial-mesenchymal transition (EMT) in lens epithelial cells, which is associated with cellular fibrosis and cataract progression [28]. Yamakuchi et al. found that overexpression of *miR-22* directly suppresses the expression of HIF-1α, impacting the production of its downstream signaling proteins, such as vascular endothelial growth factor, which may contribute to angiogenesis and responses to oxidative stress [29]. Additionally, *miR-22-3p* exhibits an anti-fibrotic effect by targeting histone deacetylase 6 (HDAC6), a functional protein involved in EMT development, activated under hypoxic conditions in lens epithelial cells. Wang et al. demonstrated that overexpression of *miR-22-3p* directly suppresses HDAC6 expression, thereby enhancing the acetylation and stabilization of α-tubulin in the cytoskeleton of lens epithelial cells, which inhibits EMT [30].

#### *miR-22-3p* and ocular cell survival

Downregulation of *miR-22-3p* in damaged lens epithelial cells and fibrotic cataract tissues directly correlates with the pathological progression of cataracts and enhanced lens fibrosis [30, 31]. Wang et al. introduced the *miR-22-3p*/HDAC6 regulatory axis as a functional pathway in the pathogenesis of fibrotic cataracts based on their study of human lens epithelial cells [30].

### Neuroprotective effects of miR-22-3p in retinal and non-lens neuronal models (indirect evidence)

The neuroprotective effects of *miR-22-3p* have been investigated across various central nervous system models, including retinal ganglion cells, the optic nerve, and retinal pigment epithelium. Although these findings do not provide direct evidence for cataract treatment, they offer indirect support for the broader neuroprotective significance of *miR-22-3p*, particularly within the neuronal components of the visual system. Xia et al. demonstrated in a mouse model of Alzheimer’s disease that *miR-22-3p* exerts neuroprotective effects by reducing apoptosis in neuronal cells. It achieves this by directly targeting SRY-related high-mobility group box 9 (Sox9) and decreasing beta-amyloid formation through the Nuclear Factor-κB (NF-κB) signaling pathway [20]. In the context of ocular diseases, particularly cataracts, *miR-22-3p* has emerged as a crucial modulator of anti-fibrotic and neuroprotective functions. It achieves these effects by targeting Histone lysine demethylase 6B (KDM6B) and Bone morphogenetic protein 2 (BMP2), which are primarily associated with inhibiting apoptosis and enhancing neuronal cell survival through the modulation of inflammatory pathways and the balance between oxidative and anti-oxidative processes [32]. Another neuroprotective mechanism of *miR-22-3p* involves targeting mitogen-activated protein 3 kinase 12 (MAP3K12), which suppresses the mitogen-activated protein kinase (MAPK) signaling pathway and inhibits neuronal apoptosis by reducing the expression of pro-apoptotic proteins BAX and caspase-3 [33]. Xu et al. investigated the neuroprotective effects of *miR-22* in a retinal ischemia/reperfusion injury model and found that *miR-22* selectively promotes the activation of the Extracellular signal-regulated kinases 1/2 (Erk1/2) signaling pathway in retinal ganglion cells [23]. Although Xu et al.’s study focused on a retinal ischemia/reperfusion model rather than a cataract model, it indirectly supports the feasibility of delivering *miR-22-3p* to ocular tissues and activating neuroprotective pathways, which may be adapted in the future for lens-targeted strategies.

## Therapeutic potential and delivery strategies

Given the molecular and neuroprotective significance of *miR-22-3p*, it emerges as an ideal candidate for treating ocular diseases, particularly cataracts. It is essential to distinguish between the mechanisms directly demonstrated in lens epithelial cells and cataract models, and those extrapolated from retinal or optic nerve injury studies [10, 33]. In this review, the proposed benefits of *miR-22-3p*-enriched SC-EVs for cataracts are primarily based on lens-specific evidence (e.g., *miR-22-3p* targeting KLF6 and HDAC6, modulation of oxidative stress and fibrosis in lens epithelium), while retinal and neuro-ophthalmic studies (e.g., MAPK-dependent neuroprotection of retinal ganglion cells) contextualize the broader neuroprotective potential of *miR-22-3p* and should be interpreted as mechanistically indirect supportive evidence. An additional critical issue is the development of effective strategies for delivering *miR-22-3p* into damaged ocular tissue, ensuring protection against degradation and facilitating targeted uptake by lens epithelial and retinal cells. Delivery methods can be implemented directly in *in vitro* studies using reagents such as lipofectamine, or through nanocarriers like liposomes, tetrahedral framework nucleic acids (tFNAs), and SC-EVs targeted towards the tissue of interest [23, 34–36].

Xu et al. studied an engineered *miR-22-3p*-enriched nanostructure, namely tFNAs, and identified enhanced uptake efficiency in retinal ganglion cells, along with synergistic effects on the activation of neuroprotective pathways while maintaining the stability of ocular environments devoid of toxic side effects [23]. Yu et al. proposed a novel therapeutic approach for protecting retinal ganglion cells in glaucoma by investigating the mechanism and therapeutic effects of overexpressing *miR-22-3p* in a mouse model of glaucoma using *miR-22-3p*-loaded MSC-EVs [33]. Preclinical studies in retinal and optic nerve injury models have indicated that *miR-22-3p* can improve retinal function, reduce apoptosis, and mitigate oxidative damage. Therefore, the targeted delivery of *miR-22-3p*-enriched EVs could represent a promising therapeutic strategy to complement existing approaches aimed at preserving lens clarity and preventing vision loss. However, no studies have directly examined its effects in cataract-specific *in vivo* models.

## Real-world challenges in SC-EVs therapy in clinic

Despite the advantages of employing SC-EVs, particularly *miR-22-3p*-enriched SC-EVs, several challenges highlight the limitations of using these nanovesicles in clinical settings:

### Challenges associated with the lake of standard methos for isolations, purifications and qualification of SC-EVs

For therapeutic applications, the isolation, purification, and qualification of SC-EVs are critical steps to ensure that these vesicles possess high purity, functional integrity, and bioavailability for drug delivery. The most common isolation methods include ultracentrifugation, size-exclusion chromatography, polymer-based precipitation methods, and immunocapture techniques. Ultracentrifugation remains the conventional isolation method; however, it poses risks of contamination and potential damage to isolated EVs, thereby limiting its clinical applicability. Modifications such as ultrafiltration or size-exclusion chromatography can enhance ultracentrifugation.

Polymer-based precipitation methods utilizing chemical components like polyethylene glycol offer alternative isolation strategies without requiring advanced instrumentation, making them relatively suitable for diagnostic applications. Nonetheless, contamination remains a significant limitation in these therapeutic approaches. Immunoaffinity capture methods, on the other hand, are specific isolation techniques that utilize antibodies against particular surface antigens on EVs [33].

While these methods yield high-quality EVs devoid of damage to their content, their high costs and low yields of purified counts at a clinical sample scale present major disadvantages. Currently, no gold-standard isolation protocol exists, leading to complications in reproducibility and characterization of procedures. Thus, selecting an appropriate method for isolating SC-EVs poses a significant challenge in clinical studies, ultimately affecting the safety and efficacy of treatment interventions [37].

### Challenges associated with producing miRNA-enriched SC-EVs

Methods for producing miRNA-enriched SC-EVs can be categorized into two primary approaches: endogenous and exogenous loading of the desired miRNA into the EVs (Figure 2). Endogenous loading involves modifying the parental cells to facilitate the incorporation of miRNAs, while exogenous loading entails directly introducing miRNAs into purified SC-EVs. Techniques such as transfection or transduction of parental cells using lentiviral vectors, plasmids, or synthetic RNA mimics can promote the overexpression of miRNAs, which are subsequently encapsulated in the secretory SC-EVs.

**Figure 2.**
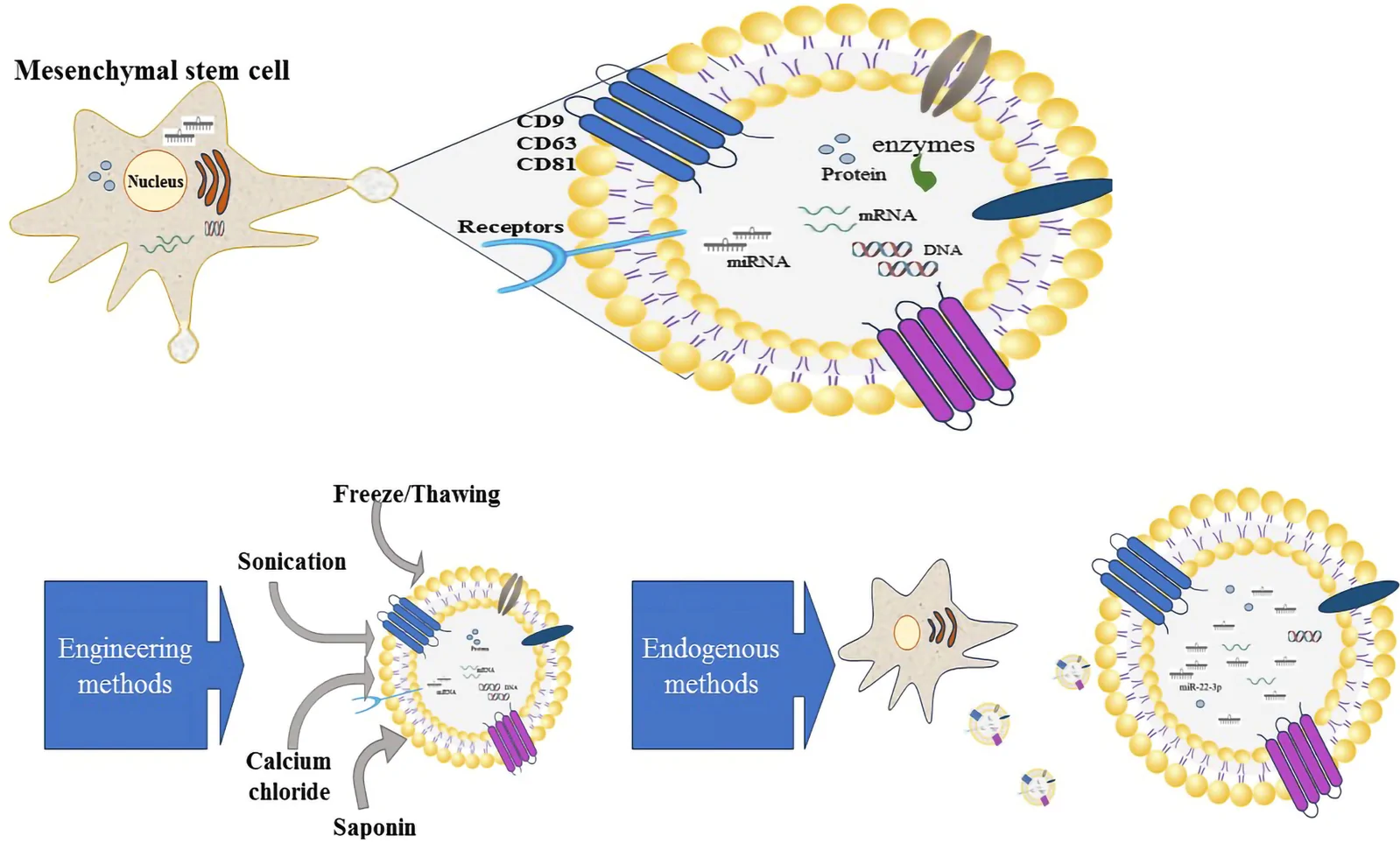
**Schematic representation of miRNA-enriched mesenchymal stem cell-derived extracellular vesicles.** This figure provides an overview of miRNA-enriched extracellular vesicles (MSC-EVs) derived from mesenchymal stem cells, highlighting their design for targeted therapeutic delivery. EVs function as nanocarriers for proteins, enzymes, mRNA, miRNA, and DNA. These nanocarriers are recognized by target cells through surface markers, including CD9, CD63, and CD81, which facilitate docking and fusion with the membranes of lens epithelial cells. Additional recognition occurs through engineered ligands specific to target cell surfaces, as well as integrins, CD44 (which interacts with hyaluronic acid on lens epithelial cells), and HSPs, which promote internalization via heparan sulfate. Endogenous methods for producing therapeutic EVs involve techniques that stimulate parental cells, such as mesenchymal stem cells, to generate targeted miRNAs or mRNAs. Conversely, exogenous engineering techniques utilize physical methods, including continuous freeze/thaw cycles, sonication, calcium chloride channel engineering, or saponin treatment, to enhance the EV membrane’s capacity for internalizing therapeutic compounds like miRNAs. **Abbreviations**: EVs, extracellular vesicles; HSPs, heat shock proteins; miRNA, microRNA; mRNA, messenger RNA; MSC-EVs, mesenchymal stem cell-derived extracellular vesicles.

Another endogenous loading method utilizes natural activators or inducing conditions, such as hypoxia, which are linked to the overexpression of specific miRNAs. Exogenous loading techniques, including electroporation, liposome-mediated loading, and sonication, facilitate the introduction of miRNAs into isolated and purified SC-EVs [38, 39]. Challenges associated with these methodologies include low efficiency and variability in the production and packaging of specific miRNA profiles, which can adversely affect pre-loading step efficacy. Post-loading challenges consist of potential miRNA aggregation, the risk of unintended damage to EVs during loading assays, and variability in loading efficiency across different SC-EVs, necessitating meticulous laboratory management and technical precision.

### Additional challenges

Further challenges in utilizing enriched *miR-22-3p* SC-EVs include insufficient data regarding the stability of developed SC-EVs in real-world applications, control of loading doses, and the cost-effectiveness of this therapeutic approach in clinical studies.

## Discussion

This review article critically examines the existing literature that supports the neuroprotective effects and functional role of *miR-22-3p* in modulating oxidative stress, fibrosis, apoptosis, and inflammation—mechanisms that contribute to cataract pathogenesis. In the context of cataract therapy, only the mechanisms that validate the functional role of *miR-22-3p* in LEC and cataract models—such as the modulation of KLF6, HDAC6, oxidative stress, and fibrosis in lens tissue—can be directly viewed as anti-cataract therapeutic insights. Conversely, mechanisms extrapolated indirectly from studies on retinal ganglion cells or other neuronal models (e.g., MAPK-dependent neuroprotection, ERK1/2 activation) should be considered as hypothesis-generating for potential combined protection of the lens and visual pathways rather than definitive proof of efficacy in cataracts [10, 18, 23, 30, 33].

Moreover, highlighting the advantages of cell-free nanocarriers, such as stem cell-derived EVs, over traditional stem cell therapy, suggests that *miR-22-3p*-enriched SC-EVs hold promise for cataract treatment. However, their clinical application is constrained by critical knowledge gaps, particularly the lack of *in vivo* studies investigating *miR-22-3p*-enriched SC-EVs in cataract animal models.

Based on the literature, the use of structurally modified EVs for cataract treatment is proposed as a potentially more effective strategy than conventional methods. Compared to intraocular lens implantation and traditional stem cell transplantation, treatment with structurally modified EVs could, in theory, be administered through various degrees of invasiveness, ranging from non-invasive to minimally invasive ocular delivery routes (e.g., topical, intracameral, or periocular), targeting the anterior segment and lens epithelial cells. For retinal or optic nerve indications, intravitreal routes may be considered, although these fall outside the primary scope of cataract therapy discussed herein. Existing preclinical studies suggest that the efficacy of EV-based treatment in cataracts may be enhanced when EVs are enriched with *miR-22-3p*.

As previously noted, *in vitro* studies and non-cataract models of the mechanistic function of *miR-22-3p* demonstrate inhibition of oxidative damage, inflammation, fibrosis, and apoptosis. However, these findings require validation through *in vivo* studies on cataract models [10, 24, 25]. The mechanisms summarized in Figure 1 illustrate pathways where *miR-22-3p* has been shown to play a direct role in LEC and cataract models (e.g., inhibition of epithelial-mesenchymal transition and fibrosis via HDAC6, modulation of KLF6-related apoptosis, antioxidant, and anti-inflammatory responses in LEC), alongside neuroprotective pathways that have been characterized indirectly in retinal ganglion cells, the optic nerve, and non-lens neuronal models (e.g., regulation of MAPK, SOX9/NF-κB, and ERK1/2 signaling). In cataract therapy, only lens-based mechanisms directly support anti-cataract effects, while retinal and neuronal data suggest additional potential benefits for the broader ocular neuroaxis.

Lens-based mechanisms include the inhibition of epithelial-mesenchymal transition and anti-fibrotic effects through the modulation of α-tubulin acetylation by targeting HDAC6 activity, thereby preventing epithelial-mesenchymal transition and lens fibrosis; regulation of antioxidant defenses and autophagy via the KDM6B/BMP2/bone marrow failure (BMF) axis and SIRT1/FOXO3, as well as KLF6; and the anti-inflammatory function of *miR-22-3p* by downregulating NLRP3 inflammasome and NF-κB, which effectively improves inflammatory responses in lens epithelial cells [10, 19, 25, 30, 36]. Additionally, *miR-22-3p* may exert anti-apoptotic effects by targeting pro-survival signaling pathways, such as SOX9/NF-κB, PTEN, and Wnt/β-catenin pathways [20, 35].

Concerns regarding the clinical application of this therapeutic approach necessitate further investigation. For instance, although KLF6 is recognized as a molecular target of *miR-22-3p* in the regulation of apoptosis in diabetic cataracts [10], we found no information about changes in its downstream targets and pathways. Similarly, the MAPK signaling pathway, identified as a target of *miR-22-3p*, modulates its neuroprotective effects in retinal ganglion cells [33]. Future studies should be designed to elucidate the downstream targets of *miR-22-3p* within its associated cellular pathways to test the hypotheses generated from the current body of preclinical evidence.

Integrating multi-omics approaches in diabetic cataracts and examining the differentiation profile following intervention with *miR-22-3p* could enhance our understanding and identification of molecular targets of *miR-22-3p*. Furthermore, the therapeutic effects of *miR-22-3p*-enriched SC-EVs require validation both *in vitro* and *in vivo*. Future studies should investigate varying doses of *miR-22-3p*-enriched SC-EVs and implement longitudinal follow-up. Despite promising findings regarding this therapeutic approach, several challenges remain unresolved, including the standardization of SC-EV isolation, characterization, optimization of loading doses and procedures for *miR-22-3p*into purified SC-EVs, and examination of these hypotheses both *in vitro* and *in vivo*, alongside monitoring treatment outcomes in clinical trials. Additionally, we found no studies comparing the efficacy of different SC-EV delivery methods as therapeutic strategies for ocular diseases. Future research should focus on identifying the most efficient delivery method and optimizing SC-EV engineering techniques for producing *miR-22-3p*-enriched SC-EVs.

## Conclusion

By integrating insights into the molecular mechanisms and pathways involved in cataract pathogenesis, the regulatory functions of *miR-22-3p* in modulating key cellular processes, and the therapeutic potential of stem cell-derived EVs in ocular diseases, this review proposes *miR-22-3p*-enriched SC-EVs as a promising treatment for cataracts. Future research should prioritize standardizing EV isolation, conducting dose-determining studies, and employing rigorous *in vivo* cataract models to ascertain the efficacy of miRNA-enriched SC-EV therapy compared to conventional surgical or pharmaceutical approaches, ultimately aiming to reduce the global burden of cataracts and associated vision loss.

## Data Availability

Data sharing is not applicable to this article as no new data were created or analyzed in this study.
